# Doxazosin zur Behandlung posttraumatischer Albträume ergänzend zu traumafokussierter kognitiver Verhaltenstherapie

**DOI:** 10.1007/s00115-026-01941-y

**Published:** 2026-02-09

**Authors:** Nikola Schoofs, Philipp Trenkmann, Kristina Meyer, Kathlen Priebe, Felix Wülfing

**Affiliations:** 1 Klinik für Psychiatrie und Psychotherapie, Charité Campus Mitte, Charité – Universitätsmedizin Berlin, Berlin, Deutschland; 2 Psychiatrische Universitätsklinik der Charité im St. Hedwig-Krankenhaus, Charité – Universitätsmedizin Berlin, Große Hamburger Str. 5–11, 10115 Berlin, Deutschland; 3 Institut für Medizinische Psychologie, Charité Campus Mitte, Charité – Universitätsmedizin Berlin, Berlin, Deutschland

## Hintergrund

Schlafstörungen werden von 70–90 % der Personen mit posttraumatischer Belastungsstörung (PTBS) berichtet [5] und bis zu 75 % der Betroffenen leiden an Albträumen [4]. Die Studienlage zum Einsatz von Psychopharmakotherapien in dieser Indikation ist uneinheitlich. Für Prazosin (α-1-Antagonist) besteht die meiste Evidenz, diese Substanz ist in Deutschland jedoch nur über die internationale Apotheke erhältlich und die Studienlage ist unklar [11]. Doxazosin, ein α‑1-Antagonist mit längerer Halbwertszeit, ist eine Alternative zu Prazosin und wird teilweise zur Off-label-Behandlung von Albträumen eingesetzt. Vor allem der Forschungsstand zu Kombinationsbehandlungen der PTBS mit Psychotherapie und Pharmakotherapie ist laut S3-Leitlinie Posttraumatische Belastungsstörung [10] unbefriedigend. Das Ziel unserer Studie war es daher, die Wirksamkeit von Doxazosin als Ergänzung zu traumafokussierter kognitiver Verhaltenstherapie zu ermitteln.

## Methodik

Wir führten eine retrospektive Analyse von 100 Patient:innen durch, die zwischen dem 01.06.2020 und dem 28.09.2023 ein geplantes 8‑wöchiges traumafokussiertes tagesklinisches Behandlungsprogramm (TF-KVT) absolvierten. Die PTBS-Symptomatik wurde mittels International Trauma Questionnaire (ITQ; [2]) in der ersten Behandlungswoche (vor Beginn der Behandlung) sowie in der letzten Behandlungswoche (zum Ende der Behandlung) erfasst. Um Schlafstörungen und Albträume zu erfassen, wurden die Items 2 und 20 aus der Posttraumatic Stress Disorder Checklist (PCL-5) extrahiert [6]. Datensätze wurden eingeschlossen, wenn sie einen Wert von ≥ 2 bei dem Item Albträume in der PCL‑5 enthielten.

Wir identifizierten 39 Patient:innen, die zusätzlich zur TF-KVT Doxazosin in der Indikation Albtraumbehandlung erhielten. Mittels Propensity Score Matching anhand von Baseline-Variablen (Albträume, Schlafstörungen, PTBS-Score, Depressivität und Lebenszufriedenheit sowie Alter und Geschlecht) wurde aus der Gruppe der Patient:innen, die ausschließlich eine TF-KVT erhalten hatten, eine Kontrollgruppe (*N* = 33) ausgewählt.

Um den Effekt der Behandlung (Prä-Post-Vergleich) für die Experimental- (KVT + Doxazosin) und Kontrollgruppe (KVT − Doxazosin) zu ermitteln, wurde für die beiden abhängigen Variablen Albträume und Schlafstörungen je eine Varianzanalyse (ANOVA) mit Messwiederholung im 2 × 2-Design (Zeitpunkt: prä vs. post; Gruppe: KVT allein vs. KVT + Doxazosin) durchgeführt. Die Personen, die Doxazosin erhielten, wurden in eine Gruppe mit hoher (> 4 mg) und niedriger Dosis (≤ 4 mg) eingeteilt. In der Doxazosin-Gruppe wurde eine zusätzliche 2 × 2-ANOVA mit Messwiederholung (prä vs. post, hoch vs. niedrig dosiert) durchgeführt, um den Effekt der Dosis auf die Symptomverbesserung zu erfassen.

Die Datenanalyse erfolgte mit SPSS 28 (IBM®). Das Signifikanzniveau wurde auf *p* < 0,05 festgelegt. Ein positives Votum der Ethikkommission der Charité lag vor (EA1/022/21).

## Ergebnisse

### Beschreibung der Stichprobe

Die Experimentalgruppe (EG) bestand aus 39 Personen (28 weiblich, 10 männlich, 1 divers). Der Altersdurchschnitt lag bei 38,3 Jahren mit einer Spanne von 18 bis 60 Jahren. Die Kontrollgruppe (KG) bestand aus insgesamt 33 Personen (28 weiblich, 4 männlich, 1 divers). Das durchschnittliche Alter lag bei 38,03 Jahren mit einer Altersspanne von 21 bis 61 Jahren.

Das traumatische Ereignis war meistens sexualisierte Gewalt (46,2 % in der EG; 48,5 % in der KG). In beiden Gruppen war die Häufigkeit von Gewalterfahrungen ähnlich: 50 % der EG berichteten von multiplen Traumata und 80–85 % von interpersoneller Gewalt, in der KG waren es 60 und 85–90 %.

Weitere demographische Daten der Stichprobe finden sich in Tab. 1.

**Tab. 1:** Deskriptive Beschreibung der Stichprobe

	Experimentalgruppe *N* = 39	Kontrollgruppe *N* = 33	*p*
	*N* (%)	*N* (%)	
*Geschlecht*			*0,352*
Männlich	10 (25,6)	4 (12,1)	**–**
Weiblich	28 (71,8)	28 (84,8)	
Divers	1 (2,6)	1 (3)	
*Alter*			*0,603*
Durchschnitt	38,23	38,03	**–**
Min	18	21	
Max	60	61	
*Familienstand*			*0,656*
Ledig	22 (56,4)	21 (63,6)	**–**
Verheiratet	6 (15,4)	6 (18,2)	
Lebensgemeinschaft	4 (10,3)	2 (6,1)	
Geschieden	7 (17,9)	4 (9,1)	
*Höchster Schulabschluss*			*0,814*
Kein Abschluss	2 (5,1)	2 (5,4)	**–**
Noch in der Schule	2 (5,1)	0 (0)	
Hauptschulabschluss	7 (17,9)	5 (15,2)	
Fachoberschulreife	9 (23,1)	7 (21,2)	
Abitur	17 (43,6)	17 (51,5)	
Sonstige	2 (5,1)	1 (3,0)	
*Höchster Berufsabschluss*			*0,600*
Keine	11 (28,2)	8 (24,2)	**–**
Lehre	11 (28,2)	9 (27,3)	
Hochschule	12 (30,8)	8 (24,2)	
In Ausbildung	4 (4,0)	3 (9,1)	
Sonstige	1 (2,6)	4 (12,1)	
*Umfang der Berufstätigkeit*			*0,546*
Vollzeit	11 (28,2)	9 (27,3)	**–**
Teilzeit	5 (12,8)	6 (18,2)	
Gelegentlich	1 (2,6)	3 (9,1)	
Nicht berufstätig	22 (56,4)	15 (45,5)	
*Psychotherapeutische/psychiatrische Vorbehandlung*			*0,456*
Ja	36 (92,3)	28 (84,8)	**–**
Nein	3 (7,7)	5 (15,2)	
*Art des Traumas*			*0,645*
Sexuelle Gewalt	18 (46,2)	16 (48,5)	**–**
Körperlicher Angriff	6 (15,4)	4 (12,1)	
Suizid/Tod Angehöriger	0 (0)	2 (6,1)	
Unfall	2 (5,1)	1 (3,0)	
Raubüberfall	1 (2,6)	1 (3,0)	
Vernachlässigung Kindheit	6 (15,4)	2 (6,1)	
Psychischer Missbrauch	2 (5,1)	1 (3,0)	
Keine Angabe	4 (10,3)	6 (18,2)	

Die durchschnittliche Dosis von Doxazosin lag bei 4,92 mg (0–10 mg). 18 Personen wurden der Hochdosisgruppe zugeordnet (> 4 mg), während 21 der Niedrigdosisgruppe (≤ 4 mg) zugeordnet wurden. Signifikante Unterschiede in Bezug auf die Einnahme sonstiger Medikamente (Psychopharmaka) bestanden nicht (Tab. 2).

**Tab. 2:** Begleitende Psychopharmakotherapie

Medikamentengruppe	Experimentalgruppe *N* = 39	Kontrollgruppe *N* = 33	*p*
	*N* (%)	*N* (%)	
Antidepressiva	27 (69,2)	20 (60,6)	0,444
Antipsychotika	15 (38,4)	9 (27,3)	0,316
Stimmungsstabilisierer	4 (10,3)	2 (6,1)	0,285

Prüfung mittels χ^2^-Test bzw. Fischer-Test bei *n* < 5

Insgesamt handelte es sich um eine Kohorte mit einer hohen Symptomlast der PTBS (Tab. 3).

**Tab. 3:** Symptomatik Prä- und Post-Intervention über beide Gruppen

Variable	Gruppe	Prä	Post			
		*M (SD)*	*M (SD)*	*t*	*p*	Cohen’s *d*
Albträume (PCL-5)	KVT − Doxazosin	3,18 (0,92)	1,91 (1,44)	−5,41	< 0,001**	−0,94
	KVT + Doxazosin	3,13 (0,92)	1,97 (1,27)	−5,13	< 0,001**	−0,82
Schlafstörung (PCL-5)	KVT − Doxazosin	3,24 (0,97)	2,24 (1,39)	−4,06	< 0,001**	−0,71
	KVT + Doxazosin	3,26 (0,94)	2,67 (1,16)	−3,30	0,001**	−0,53
ITQ-Score	KVT − Doxazosin	34,76 (5,50)	23,48 (9,39)	−6,27	< 0,001**	−1,09
	KVT + Doxazosin	35,62 (5,85)	25,82 (8,29)	−7,03	< 0,001**	−1,13

*PCL‑5* Postraumatic Stress Disorder Checklist (0–4), *ITQ* International Trauma Questionnaire (0–48)

### Behandlungseffekt

Mittels ANOVA mit Messwiederholung fanden wir signifikante Haupteffekte der Zeit auf die verschiedenen abhängigen Variablen (*p* < 0,001 in allen Modellen), aber keine signifikanten Haupteffekte der Gruppe (KG vs. EG; *p* > 0,32) und keine signifikanten Interaktionen zwischen Zeitpunkt und Gruppe (*p* > 0,17). Es zeigen sich also zwischen der KG und der EG keine Unterschiede in Bezug auf Albträume und Schlafstörungen. Ebenso ist kein additiver Effekt durch eine höhere verglichen mit einer niedrigeren Dosis Doxazosin zu sehen (Haupteffekte: *p* > 0,52, Interaktionen: *p* > 0,21).

## Diskussion

In unserer retrospektiven Datenanalyse untersuchten wir die Wirksamkeit einer zusätzlichen Behandlung von Doxazosin und traumafokussierter KVT gegenüber KVT ohne zusätzliche Gabe von Doxazosin bei PTBS-Betroffenen mit klinisch relevanten Albträumen.

Insgesamt zeigte sich nach der traumafokussierten Therapie eine Reduktion der PTBS-Symptomatik und eine Reduktion von Albträumen (große Effekte) sowie eine Reduktion von Schlafstörungen (mittlerer Effekt). Eine zusätzliche Gabe von Doxazosin hatte keinen zusätzlichen Effekt auf die von uns untersuchten Endpunkte.

Die Datenlage zur Behandlung der PTBS mit Doxazosin ist insgesamt klein. Eine Pilot-RCT (*N* = 8; [8]), eine kleine unverblindete Studie [3] und eine retrospektive Datenanalyse [1] geben Hinweise auf eine mögliche Wirksamkeit. Die einzige größere RCT (*n* = 141) untersuchte Veteranen mit PTBS und komorbider Alkoholabhängigkeit und konnte keine Überlegenheit von Doxazosin gegenüber Placebo feststellen. Albträume und Schlafstörungen wurden allerdings nicht gesondert untersucht [1].

Bislang untersuchte keine Studie gezielt eine Kombinationstherapie aus traumafokussierter Psychotherapie und Doxazosin.

Traumafokussierte Psychotherapie ist der Goldstandard in der Behandlung der PTBS [10]. Wir vermuten, dass die traumafokussierte KVT in unserer Stichprobe den größten Teil der Symptomreduktion erklärt. In der klinischen Praxis, vor allem in (teil)stationären Settings wie auch in unserem Fall, werden Individuen mit PTBS jedoch regelmäßig mit multimodalen Behandlungskonzepten behandelt, welche sowohl psychotherapeutische als auch pharmakologische und adjuvante Interventionen enthalten, obwohl Daten zu diesen Kombinationsbehandlungen fehlen.

Es ist anzumerken, dass hohe Standardabweichungen der Symptomreduktion für alle untersuchten Parameter festgestellt wurden. Dieses Ergebnis könnte darauf hinweisen, dass die Teilnehmenden in sehr unterschiedlichem Ausmaß von der Behandlung profitierten. Aufgrund der recht kleinen Stichprobengröße ist die Interpretierbarkeit der Ergebnisse daher eingeschränkt.

In Bezug auf die Dosierung des Doxazosin gab es in unserer Arbeit keine Unterschiede zwischen der Hoch- und Niedrigdosisgruppe. Im Gegensatz dazu fanden andere Arbeiten, dass eine höhere Doxazosindosis mit einer signifikant verstärkten Symptomreduktion verbunden war [7]. Da in anderen Studien insgesamt höhere Dosen erreicht wurden, ist es denkbar, dass in unserer Stichprobe die Dosierung insgesamt nicht ausreichend hoch war, um eine zusätzliche Wirksamkeit zu erzielen.

## Limitationen

Unsere Studie stellt eine retrospektive Datenanalyse dar und genügt somit nicht den Kriterien einer randomisiert-kontrollierten Studie. Andererseits bildet unsere Arbeit beruhend auf einer naturalistischen Stichprobe die Versorgungsrealität ab, was als Vorteil unseres quasi-experimentellen Designs gewertet werden kann. Wir erfassten die Outcomes anhand von Selbstbeurteilungsfragebögen. Zudem wurden die Items für die Variablen Albträume und Schlafstörungen aus dem Fragebogen PCL‑5 extrahiert, sodass nur eine begrenzte Menge an klinischen Informationen zu diesen Parametern erfasst wurde.

Eine weitere Limitation ist, dass die begleitende Pharmakotherapie nicht standardisiert war im Rahmen des retrospektiven Designs.

## Ausblick

Unsere Daten zeigen keine Hinweise auf einen additiven Nutzen von Doxazosin gegenüber einer traumafokussierten KVT. Aufgrund der insgesamt unzureichenden Datenlage sind weitere randomisiert-kontrollierte Studien zur Wirksamkeit von Doxazosin in Kombination mit KVT zur Behandlung posttraumatischer Schlafstörungen wünschenswert.

## Fazit für die Praxis

- Nach tagesklinischer traumafokussierter kognitiver Verhaltenstherapie zeigte sich eine Reduktion der allgemeinen PTBS-Symptomatik sowie der posttraumatischen Albträume und Schlafstörungen.
- In unserer Stichprobe zeigten sich keine signifikanten Unterschiede zwischen traumafokussierter KVT mit und ohne zusätzliche Gabe von Doxazosin (durchschnittlich 4,9 mg).
- Hochdosis- (>4 mg Doxazosin) und Niedrigdosisgruppe (≤ 4 mg Doxazosin) unterschieden sich nicht signifikant in Bezug auf die Reduktion von PTBS-Symptomatik sowie Albträumen und Schlafstörungen.
